# Invasion genomics of lionfish in the Mediterranean Sea

**DOI:** 10.1002/ece3.11087

**Published:** 2024-03-05

**Authors:** Giacomo Bernardi, Ernesto Azzurro, Michel Bariche, Carlos Jimenez, Stefanos Kalogirou, Periklis Kleitou

**Affiliations:** ^1^ Department of Ecology and Evolutionary Biology University of California Santa Cruz Santa Cruz California USA; ^2^ CNR‐IRBIM, National Research Council Institute of Biological Resources and Marine Biotechnologies Ancona Italy; ^3^ Zoological Station A. Dohrn Naples Italy; ^4^ Department of Biology American University of Beirut Beirut Lebanon; ^5^ Enalia Physis Environmental Research Centre (ENALIA) Nicosia Cyprus; ^6^ The Cyprus Institute Energy Environment and Water Research Center Nicosia Cyprus; ^7^ Hellenic Centre for Marine Research, Institute for Marine Biological Resources and Inland Waters Hydrobiological Station of Rhodes Rhodes Greece; ^8^ Marine & Environmental Research (MER) Lab Limassol Cyprus; ^9^ School of Biological and Marine Sciences University of Plymouth Plymouth UK

**Keywords:** bioinvasion, lessepsian migrants, mediterranean, Pterois miles

## Abstract

The rate of biological invasions is steadily increasing, with major ecological and economic impacts accounting for billions of dollars in damage as a result. One spectacular example is the western Atlantic invasion by lionfishes. In the Mediterranean Sea, invasions from the Red Sea via the Suez Canal (termed Lessepsian invasions) comprise more than 100 fish species, including a recent invasion by lionfish. In light of the devastating effects of lionfish in the Caribbean Sea, understanding the dynamics of Mediterranean lionfish invasion is crucial. The Lessepsian lionfish invasion started in 2012, and rapidly spread to the central Mediterranean. Here, we used thousands of RAD seq genomic markers to study the population dynamics of this invasion. While we did not find a reduction in genetic diversity between source (Red Sea) and invasive (Mediterranean) populations (i.e., bottleneck effects), we found evidence of population structure within the invasive range in the Mediterranean Sea. We found that loci that are potentially under selection may play an important role in invasion success (in particular, genes involved in osmoregulation and fin spine sizes). Genomic approaches proved powerful in examining the ecological and evolutionary patterns of successful invaders and may be used as tools to understand and potentially mitigate future invasions.

## Graphical abstract

Lionfish are well‐known for having invaded the Caribbean with devastating ecological effects. Here we study the genomics of a recent invasion of lionfish in the Mediterranean, coming from the Red Sea via the Suez Canal. We found that genetic diversity has not declined during the invasion phase, and some genes potentially under selection may have contributed to the invasion success.

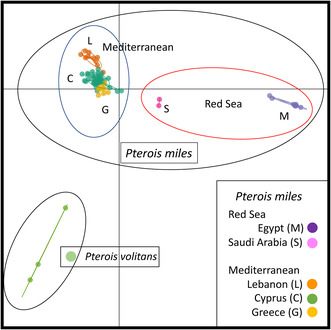

## 1 INTRODUCTION

Biological invasions are widely regarded as a major threat to native ecosystems and to global biodiversity (Bellard et al., 2016; Kumschick et al., 2015). The increase in global transport combined with major anthropogenic changes have favored the introduction of thousands of non‐indigenous species (NIS) worldwide, some of them establishing invasive populations that result in ecological devastation (Fristoe et al., 2021), and annual losses amounting to billions of dollars to local economies (Diagne et al., 2021). The aquatic environment is not immune to the phenomenon. The introduction of the Zebra mussel *Dreissena polymorpha* in the Great Lakes (Sakai et al., 2001), the population explosion of the warty comb jelly *Mnemiopsis leidyi* in the Eurasian seas (Shiganova et al., 2019), and the invasion of the western Atlantic by lionfish *Pterois volitans/miles* (Hixon et al., 2016) are just a few among the many examples of major economic and ecological impacts.

Red Lionfish, *Pterois volitans*, naturally occur in the tropical Pacific Ocean, while the congeneric Common Lionfish, *Pterois miles*, is restricted to the tropical Indian Ocean and the Red Sea, with a narrow overlap in the Indonesian region (Kulbicki et al., 2012). The introduction of lionfish in western Atlantic waters is most likely due to the ornamental fish trade, and it is generally acknowledged that a small founder population originated in south Florida after multiple aquarium releases (Selwyn et al., 2017). Lionfishes, primarily *P. volitans*, and to a lesser extent *P. miles*, have rapidly spread, since the late 1980's, throughout the Western North‐Atlantic, the Caribbean Sea, the Gulf of Mexico, and Brazil, reaching high densities and inflicting important damages to ecological services (Ferreira et al., 2015; Green et al., 2012; Hixon et al., 2016; Luiz et al., 2021; Ruttenberg et al., 2012). This invasion has been disastrous as the invaders directly compete for food resources with native predators and intensively feed on native reef fishes (Rocha et al., 2015; Tornabene & Baldwin, 2017), and at the same time are rarely preyed upon (Maljković et al., 2008; Mumby et al., 2011). The success of lionfish can be related to several biotic and abiotic factors (Côté & Smith, 2018). At the genetic level, little diversity is present in the invading populations (Bors et al., 2019; Burford Reiskind et al., 2019; Pérez‐Portela et al., 2018; Whitaker & Janosik, 2020), which is consistent with relatively few founding invaders, a common pattern in invasion biology (Roman & Darling, 2007; Sakai et al., 2001). It was also shown that observed heterozygosity gradually diminishes as one moves further away from the invasion source (Bors et al., 2019). More intriguing is the idea that invading individuals may have been boosted by hybridization events that would give them additional invasion potential via heterosis (hybrid vigor) (Whitaker & Janosik, 2020; Wilcox et al., 2018).

Recently, the common lionfish, *P. miles*, has started invading the Mediterranean Sea. There, the situation is rather different than for the Caribbean. The Mediterranean region is a hotspot for biological invasions, principally due to the opening of the Suez Canal, in 1869 (Tiralongo et al., 2022). To date, more than 100 fish species have immigrated across this route, from the Red Sea into the Mediterranean, starting a phenomenon referred to as Lessepsian bioinvasion (after the engineer of the Suez Canal, Ferdinand de Lesseps) (Por, 1978). In the early years after the opening of the Canal, few migrations were observed, probably due to the presence of natural barriers to migration, including the Bitter Lakes, a natural hypersaline barrier, and the freshwater plume of the Nile River which flows in front of the Mediterranean opening of the Suez Canal. The construction of the Aswan dam, in 1960, which reduces the Nile outflow, and the widening of the Canal itself, which dilutes the Bitter Lakes (through increased tidal range), dampened those natural barriers, resulting in an increase in migration rate of Lessepsian bioinvaders.

**FIGURE 1:**
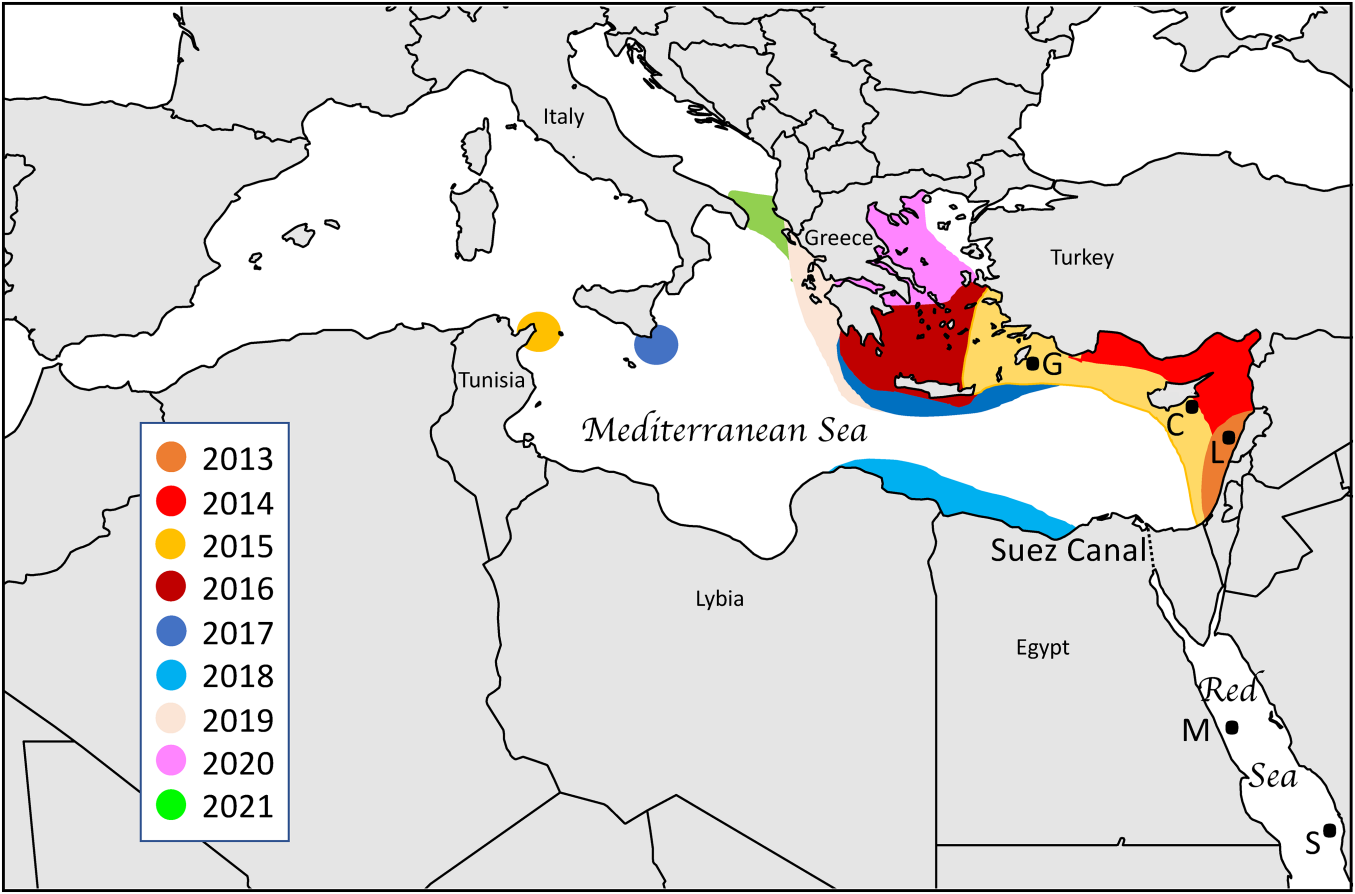
Map of the study region showing sampling locations used in this study: G: Greece, C: Cyprus, L: Lebanon, M: Marsa Alam, Egypt, S: Shibb Masturah, Saudi Arabia. Colored contours on the map indicate the extent of the invasion as it progressed by year.

A single lionfish, *P. miles*, was first recorded in 1991, along the eastern coast of the Mediterranean basin (Golani & Sonin, 1992), yet no additional individuals were observed until 2012 (Bariche et al., 2013). Given the conspicuous morphology of lionfishes, which are unlike any native species, and the significant human presence in the eastern Mediterranean Sea, it is likely the first observation led to an unsuccessful establishment of the Common Lionfish, rather than an oversight of additional undetected invaders. The lack of connectivity among potential lionfish habitats in the Mediterranean was initially considered a major hindrance to a wide dispersion of this species (Johnston & Purkis, 2014), but the lionfish population exploded soon after its second detection in 2012 (Bariche et al., 2013, 2017; Dimitriou et al., 2019), spreading quickly to the central Mediterranean (Azzurro et al., 2017) (Figure 1). Biological characteristics of lionfish such as being an *r*‐selected species (early maturity, year‐round reproduction, fast growth, and broad niche; Fogg et al., 2017; Savva et al., 2020), combined with the lack of predators (Ulman et al., 2021), and the use of new foraging techniques toward naïve native prey (Akins et al., 2014; D'Agostino et al., 2020), favored its rapid spread and invasion into the Mediterranean Sea.

The factors that promote invasion success in Lessepsian fishes are still unclear. Most Mediterranean populations do not exhibit genetic bottlenecks that are expected from the invasion of a small number of individuals, except for very few cases such as *Fistularia commersonii*, the bluespotted cornetfish (Bernardi et al., 2010, 2016). On the other hand, mutations followed by local adaptation are unlikely to occur within the Mediterranean due to the short time most Lesspesian fishes have spent since their invasion event. Instead, at least in some cases, a large number of individuals might be entering the Mediterranean, but only preadapted ones might be able to survive in the new environment (Azzurro et al., 2022; Bernardi et al., 2016). In the Red Sea (source population), those individuals contain allelic variants that, once in the Mediterranean, give them selective advantages. As such, those alleles may be identified as under selection in genome scans. Candidate genes potentially under selection include those involved in osmoregulation since the passage from the Red Sea into the Mediterranean is hampered by a number of osmotic barriers, chiefly represented by the Bitter Lakes as discussed above (Azzurro et al., 2022; Bernardi et al., 2016). There are, therefore, expectations of genetic bottlenecks (apparently rare) and/or the presence of genes under selection that result from preadaptation to the new environment.

While signals of selection may be identified, range expansions of invading populations (when large number of invading individuals are present, or multiple invasion events occur) can display a lowering of genetic diversity with distance from the source of invasion, together with the increase of rare allele frequencies at the edge of the invasion front, due to repeated founder events through space and time, which has been termed allele surfing (Excoffier et al., 2009). Thus, the presence of large numbers of invading individuals, or multiple invading events, might result in signatures of selection or allele surfing, which are not easy to distinguish (Bors et al., 2019; Goodsman et al., 2014; Pierce et al., 2014).

In light of the devastating effects of lionfish in the Caribbean Sea, understanding the dynamics of Mediterranean lionfish invasion is crucial. For example, the possible co‐occurrence of *P. miles* and *P. volitans* in the Mediterranean Sea raises several questions regarding their relative contribution to the invasion and the potential for hybridization. In addition, the role of local adaptation and natural selection may play a very important role in the success of these invasions (Poursanidis et al., 2020).

A large gap of knowledge on the genomics of the Mediterranean lionfish invasion warrants 1. to determine the identity of these invaders and their potential hybrid status, 2. to evaluate the level of gene flow between the original and invading populations and 3. to assess neutral and potentially selected genomic regions that might confer local adaptation capabilities to the Mediterranean invaders. To achieve these goals, we used thousands of genomic markers (RAD sequences), to provide a detailed picture of the genomic background of the Mediterranean lionfish.

## 2 MATERIALS AND METHODS

### 2.1 Sampling

*Pterois volitans*, a species native to the Pacific Ocean, and *P. miles*, a species found in the Red Sea and Indian Ocean, have both been claimed to occur in the Mediterranean. We used a few samples of *P. volitans* early in the analyses, in order to ascertain that the Mediterranean population we sampled is derived from the Red Sea, as is more commonly suggested, rather than a mixture of the two potential species. A total of 69 samples of lionfish were collected by spear while free diving or scuba diving, from local spearfishermen, or by directly taking fin clips from the fish while scuba diving (methods per location are provided in Table 1). We collected 66 individuals identified as *P. miles* in the Mediterranean (Lebanon, Cyprus, and Greece) and the Red Sea (Egypt and Saudi Arabia), and three *P. volitans* individuals from Micronesia (one sample) and Cuba (two samples, an invading population outside its natural range). Samples were collected from 2014 to 2018 in the Mediterranean and from 2011 to 2019 elsewhere (Figure 1, Table 1). Fin tissues were immediately placed in 95% ethanol and kept at ambient temperature until reaching the laboratory where they were stored in freezers at −20°C.

**TABLE 1:** Sampling locations for lionfish used in this study.

*Species*	Year of collection	*N*	Na	var	poly	private	P	Obs_Het	Exp_Het	pi	Fis
Sampling site											
***Pterois miles***											
Red Sea		10	1.598	8677	5186	414	0.88795	0.16236	0.16036	0.17021	0.02171
Shibb Masturah, Saudi Arabia^1^	2014	2	1.303	5440	1648	132	0.90133	0.15653	0.12507	0.16676	0.01535
Marsa Alam, Egypt^2^	2019	8	1.568	8337	4734	575	0.88534	0.16783	0.16235	0.17417	0.01716
Mediterranean Sea		56	1.961	10,743	10,329	3491	0.86804	0.19414	0.18584	0.18774	−0.01287
Lebanon^3^	2015–2016	10	1.645	11,254	7262	1007	0.85951	0.21454	0.19516	0.20703	−0.01704
Cyprus^1,3^	2016–2018	37	1.845	10,497	8875	2657	0.86277	0.20404	0.19281	0.19579	−0.01709
Rhodes, Greece^1^	2018	9	1.569	8214	4671	670	0.87764	0.18054	0.16984	0.18057	0.00197
***Pterois volitans***											
Ulithi, Falalop, Micronesia^2^	2019	1									
Trinidad, Cuba^1^	2011	2									

*Note*: Species names, sampling locations, year of collection, and number of samples are presented in this order from left to right. Further columns correspond to: Na: Number of alleles; var: variable alleles; poly: polymorphic sites; private: number of private alleles in the population; P: major allele frequency (average); Obs Het: observed heterozygosity; Exp Het: expected heterozygosity; pi: nucleotide diversity; Fis: inbreeding coefficient. Collection methods are mentioned for each location by superscripts. These included spearing while free diving or scuba diving (1), directly taking fin clips from the fish while scuba diving (2), or obtaining samples from local spearfishermen (3).

### 2.2 DNA extraction – library preparation

DNA was extracted from 25 mg fin clips using DNeasy Blood & Tissue kits (Qiagen). Fin clips were kept overnight at 56°C in Lysis buffer and protein K, before proceeding to column extraction according to the manufacturer’s protocol. We constructed RAD libraries using a variation of the original protocol with the restriction enzyme SbfI (Baird et al., 2008; Longo & Bernardi, 2015; Miller et al., 2007, 2012). Initial genomic DNA amount for each individual was 400 ng. Libraries were physically sheared on a Covaris S2 sonicator with an intensity of 5, duty cycle of 10%, cycles/burst of 200 and a cycle time of 30 s. We carried out the final PCR amplification step in 50 μL reaction volumes with 10 amplification cycles. Ampure XP beads (Agencourt) were used for each purification step and size selection. The library was sequenced in a single lane on an Illumina HiSeq 4000 at the Vincent J. Coates Genomics Sequencing Laboratory at UC Berkeley (USA). Then, we applied Perl scripts to trim the raw reads to 92 base pairs (bp) on the 3′ end, quality filtered and demultiplex them according to the 6 bp unique barcodes. Reads with Phred scores of <33 were discarded. The barcodes and restriction site residues (6 bp) were removed from the 5′ end, and this resulted in a final sequence length of 80 bp (see Data S1).

### 2.3 RAD analysis

The program STACKS version 2.2 (Catchen et al., 2011, 2013) was used to identify orthologous sequences among individuals. In order to optimize the STACKS protocol, we followed published guidelines (Rochette & Catchen, 2017). Briefly, we first identified 14 individuals with the highest sequencing coverage and created a catalog of loci based on these samples (using the ustacks and cstacks components of STACKS). In addition, we optimized the values of the parameters *M* and *n* (*M* and *n* values were kept identical, *M* being the maximum distance (in nucleotides) allowed between stacks and *m* being the minimum depth of coverage required to create a stack), by varying them from 1 to 9 as recommended (Rochette & Catchen, 2017). We found that the optimal value for *M* and *n* was 4. We then included the remaining 52 (or 55 depending on the dataset) samples using ustacks.

### 2.4 Analysis of population structure

Using the population component in STACKS, we further filtered the dataset by retaining loci which aligned in >80% of individuals (r command, ‐r 0.8) in every population. In order to remove paralogs, we used the minor alleles function of STACKS following recommendations (min_mav 0.05). We then generated genepop files using the populations component in STACKS with the write_single_snp option (a single SNP was kept for each locus), which were converted afterward using the program PGDSpider V.2.1.0.3 (Lischer & Excoffier, 2012).

We examined genetic structure in neutral loci and outlier loci, separately, to investigate the possibility that different factors have shaped population divergence in unique patterns. Here, we define neutral loci as those that are not identified as outlier loci. This definition is not ideal; however, since most RAD loci are non‐coding, using alternative approaches to define neutrality, related to synonymous versus non‐synonymous sites for example, would be too restrictive for proper statistical analyses. Population structure was analyzed using STRUCTURE and DAPC approaches. First, structure files from the STACKS populations output were analyzed using a Bayesian approach in STRUCTURE version 2.3.4 (Pritchard et al., 2000). Ten replicate runs were performed for a range of *K* from one to seven, with 10,000 iterations as the burn‐in parameter and 100,000 iterations under the admixture model. The highest likelihood for *K* was estimated according to the Evanno method (Evanno et al., 2005) implemented in Structure Harvester (Earl & VonHoldt, 2012). Second, we performed a Discriminant Analysis of Principal Components (DAPC) (Jombart et al., 2010; Miller et al., 2020), which combines the benefits of discriminant and principal component analyses and is particularly useful to study differences between clusters (i.e., sites or populations) as it utilizes a multivariate approach to explore the entire variation in the data while minimizing that within clusters. This analysis was performed using the ADEGENET package in R (Jombart, 2008; R Core Team, 2016) using the vcf file produced by the STACKS populations output used as an input file. The algorithm find.clusters identified the plausible number of clusters by comparing Bayesian Information Criterion (BIC) values, and the cross‐validation tool xvalDapc determined the number of principal components that were retained.

### 2.5 Outlier loci

Outlier loci were identified using two packages, LOSITAN and PCADAPT (Antao et al., 2008; Privé et al., 2020). Loci identified by both approaches were kept for subsequent analyses. Loci identifiers were then placed in “blacklists” and “whitelists,” which were used to run population scripts again for neutral and outlier analyses, respectively. Although working with outliers might incorporate a series of shortfalls (Bierne et al., 2011, 2013; Lotterhos & Whitlock, 2015), in particular on expanding populations such as invading ones (Lotterhos & Whitlock, 2015), they are used to identify the potential diverging effects of selection between populations (Bernardi et al., 2016; Gaither et al., 2015; Longo & Bernardi, 2015; Stockwell et al., 2016).

### 2.6 Functional analyses

All outlier loci were compared to GenBank entries with BLAST, where *E*‐values of 0.001 and below were kept and recorded (probability of obtaining the same result by chance <0.001). When protein‐coding matching sequences were found, they were classified using KEGG (Kyoto Encyclopedia of Genes and Genomes) assignments (Kanehisa et al., 2008; Ogata et al., 1999).

## 3 RESULTS

### 3.1 Loci and polymorphism statistics

Approximately 100 million reads were produced from the sequencing of genomic DNA on one Illumina lane. Following optimization of the genotyping catalog in STACKS (Rochette & Catchen, 2017), the total number of loci was 52,218, with polymorphic loci passing all filtering criteria resulting in 15,348 SNPs. As previously mentioned, in order to avoid any biased use of non‐independent SNPs, a single SNP per locus was used for all the analyses described below.

### 3.2 *Pterois miles* and *P. volitans*

**FIGURE 2:**
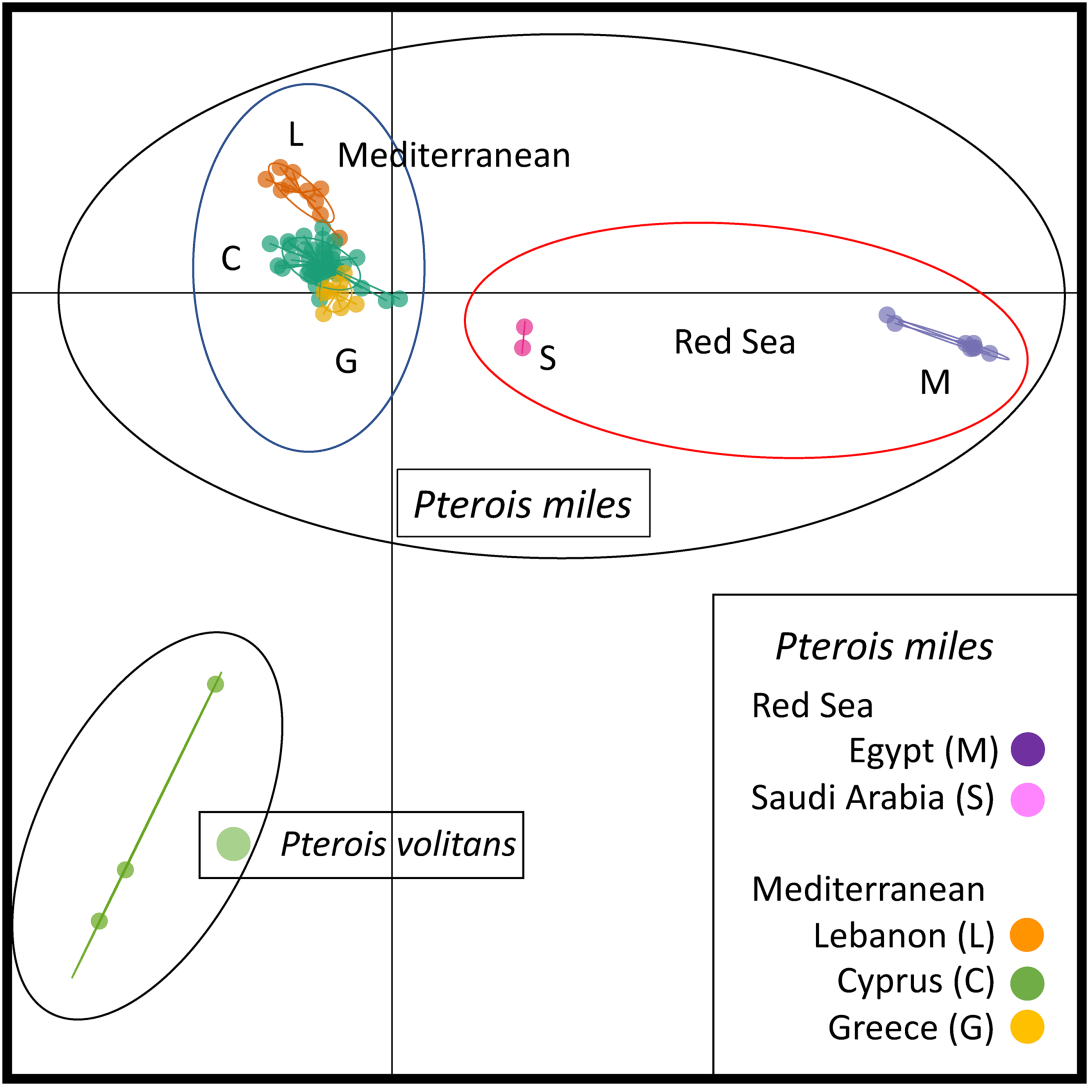
Discriminant analysis of principal components (DAPC) cluster plots of *Pterois miles* and *Pterois volitans* based on 15,348 SNPs. Plots were created in R using the adegenet package.

The occurrence of the red lionfish, *P. volitans*, has been reported from Turkey (Gürlek et al., 2016; Turan et al., 2017), indicating that our samples could potentially belong to two separate species or hybrids of the two species. Since the Mediterranean invasion of lionfishes is very recent, and *P. volitans* is very rare, introgressed individuals would be expected to be F1 hybrids, or potentially backcrosses, which would display intermediate (or at least very identifiable) genotypes compared to the pure species. Three samples were identified as *P. volitans*, one from the natural range (Micronesia), and two from the Caribbean (Cuba), grouped together in a DAPC (Figure 2). None of the samples from the Red Sea (where only *P. miles* is present) and the Mediterranean clustered with that group (Figure 2). In addition, the DAPC did not place any individual in an intermediate position between the “*P. miles* cluster” and the “*P. volitans* cluster” suggesting that none of our Mediterranean samples were hybrids of the two species (Figure 2). We, therefore, concluded that all the Red Sea and Mediterranean samples were pure *P. miles* allowing us to proceed with intraspecific analyses.

### 3.3 Population structure analyses – *P. miles*

The remainder of the analysis was done on the *P. miles* samples only (66 individuals), reducing the number of loci to 50,284. Polymorphic loci that passed all filtering criteria resulted in a total dataset of 14,293 SNPs (only one SNP per locus was used for the analyses, Table 1). While the sample numbers varied between collection localities (populations), they did not significantly correlate with the number of usable loci, average number of alleles (i.e., levels of polymorphism), and observed heterozygosity (Table 1) for each population, suggesting that performing a comparison between populations of different sizes might be appropriate. Previous studies have shown that sample sizes as small as 3 or 4 individuals provide accurate estimates of *F*_st_, provided that loci numbers are large (>3000 SNPs) (Qu et al., 2020; Willing et al., 2012). In our case, loci numbers are well above that number, suggesting that population genetics estimates obtained in this study are likely to be robust. In addition, while pairwise differences in population sample sizes may introduce biased estimates of *F*_st_ (Weir, 1979), those estimates are again made robust in current studies when very large numbers of SNPs are used to generate data (Qu et al., 2020), as was the case in this study. Nevertheless, for those populations where sample sizes are small, results must be evaluated with caution.

### 3.4 Genetic diversity

There was no significant difference between the genetic characteristics of Red Sea and Mediterranean populations, suggesting a lack of founder effect, indicating that the invasion is the result of either multiple introduction events or a large number of propagules (Bernardi et al., 2010; Sendell‐Price et al., 2021). In particular, there was no significant decrease in number of alleles, heterozygosity, or genetic diversity *π* (pi) values in Mediterranean populations compared to the Red Sea ones. For example, the genetic diversity (*π*) was 0.170 in the Red Sea and 0.188 in the Mediterranean Sea (Table 1).

For Atlantic lionfish, it was found that observed heterozygosity decreased linearly with distance from Florida (the origin of spread), and both allelic richness and expected heterozygosity remained unchanged throughout the sampled range (Bors et al., 2019). Sampling in the Mediterranean is not as vast in both numbers and geographic locations as in the Caribbean, thus precluding a statistically supported result that could be compared with what is described above. However, we also found that observed heterozygosity decreased steadily as the sampling locality was farther away from the Suez Canal (Figure S1), and both allelic richness (average number of alleles per locus) and expected heterozygosity remained steady throughout the sampled range (not shown). Again, these are numbers based on few samples; therefore, additional sampling will be necessary to buttress these trends. Similarly to the situation in the Atlantic (Bors et al., 2019), we did not observe a decrease in major allele frequency or allelic richness at the edge of the range expansion, as predicted by allele surfing. However, a potential pitfall of the approach, as described in the Atlantic invasion study, that was also based on RAD sequencing, is that allele surfing might not present in enough loci to be detectable as such. In order to properly test for allele surfing (which might still be present), would be to sequence entire genomes of individuals sampled at the edge of the range expansion.

### 3.5 Neutral loci

The analyses of population structure based on all *P. miles* orthologous loci identified by stacks resulted in a dataset of 50,284 loci comprising 14,293 usable variable SNPs. Of those, 62 were considered outliers (see below), thus we used the remaining 14,231 neutral (defined as non‐outliers) SNPs for this portion of the analysis (we also used a dataset that removed all 1037 unique outlier loci, as described below, and results remained unchanged, Figure S2). As expected, gene flow among populations was very high (average pairwise *F*_st_ = 0.044; average pairwise Φ_st_ = 0.018). It was, however, lower than a similar analysis on Atlantic lionfish, where *F*_st_ values varied between zero and a highest value of 1.2 × 10^−4^ (Bors et al., 2019). In addition, we observed slightly lower levels of gene flow between Red Sea and Mediterranean populations (average pairwise *F*_st_ = 0.055; average pairwise Φ_st_ = 0.034), than within the Mediterranean, where gene flow among populations was lowest (average pairwise *F*_st_ = 0.019; average pairwise Φ_st_ = 0.003).

**FIGURE 3:**
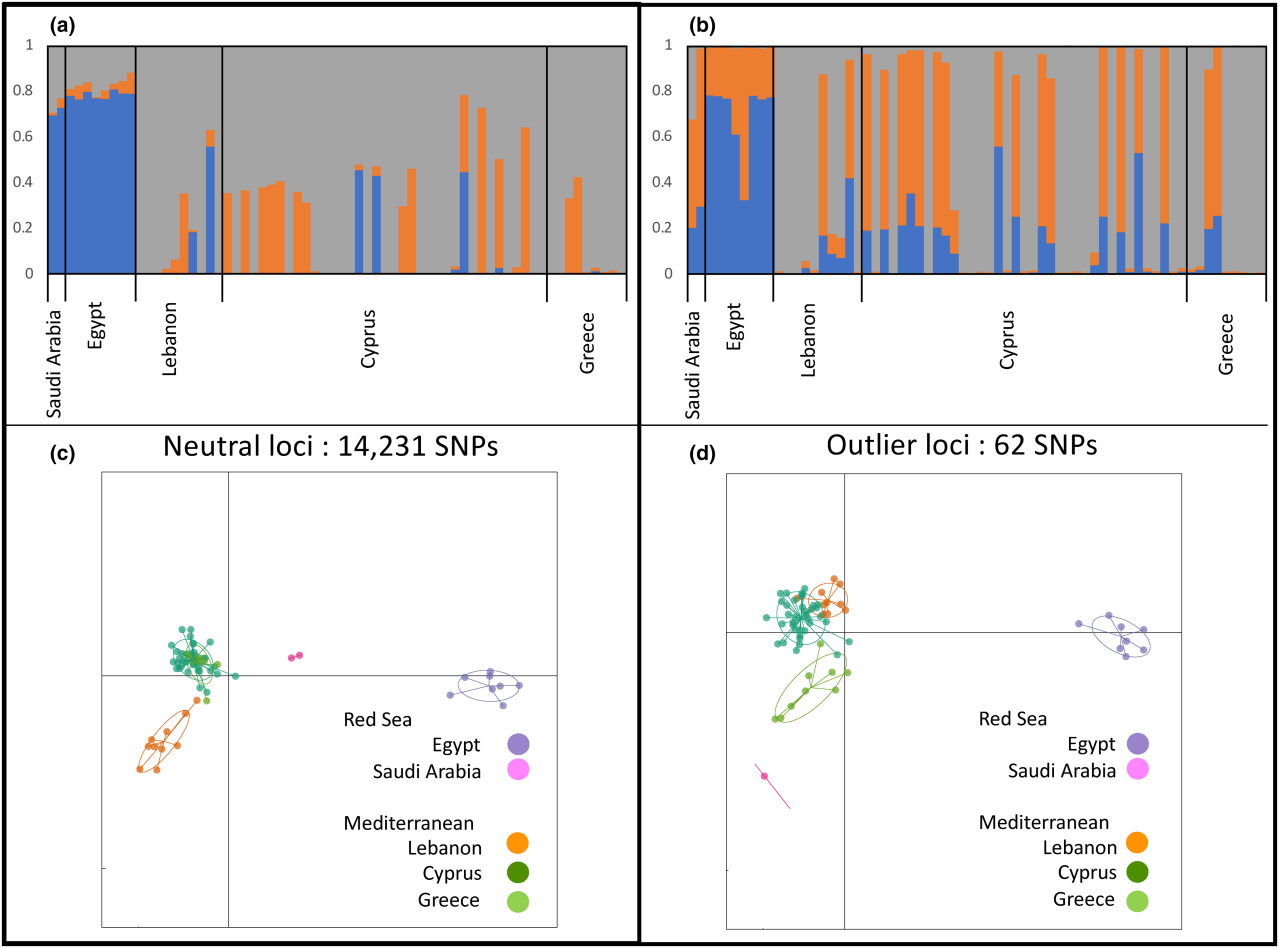
STRUCTURE and Discriminant analysis of principal components (DAPC) plots of *Pterois miles* individuals from the Red Sea and the Mediterranean based on neutral and outlier SNPs. STRUCTURE plots (*K* = 3) with Bayesian assignment of individuals (vertical bars) based on presumed neutral loci (panel a) and outlier loci, or loci suspected to be under selection (panel b). DAPC plots were created in R using the adegenet package and are shown in the bottom panels (c) and (d) with colors corresponding to sampling sites.

STRUCTURE HARVESTER suggests that our data reach the highest likelihood when clustered in three (*K* = 3) genetic groups (Figure 3a). STRUCTURE results reflect the *F*_st_ values described above, where Red Sea individuals may be distinguished from Mediterranean ones, while little genetic structure is discernible within the Mediterranean. An analysis of DAPC gave similar results, where Red Sea and Mediterranean populations belong to different clusters, but can additionally separate individuals collected in Lebanon from other Mediterranean populations (Figure 3c).

### 3.6 Outlier loci

We used two approaches to identify outlier loci, LOSITAN and PCADAPT, and then focused on those loci that were shared by both methods. In order to determine if natural selection played a role in the successful invasion of the Mediterranean by lionfishes, we partitioned the dataset in Red Sea and Mediterranean individuals as two separate populations (i.e., without distinguishing populations within these two regions). LOSITAN identified a total of 520 outlier loci, while PCADAPT identified a total of 579, for a combined total of 1037 unique loci and 62 loci shared by both approaches. We, therefore, analyzed populations based on a dataset of 62 loci that are potentially under selection (outlier loci). As expected, the average pairwise *F*_st_ among populations was higher for outlier loci (average: 0.067), and values for Φ_st_ were also higher (average: 0.071).

Fixation indexes between the Red Sea and the Mediterranean populations (*F*_st_ = 0.099, Φ_st_ = 0.130) were much higher than the fixation indexes of populations within the Mediterranean (*F*_st_ = 0.024, Φ_st_ = 0.014). This pattern was again mirrored by STRUCTURE and DAPC analyses. Indeed, the STRUCTURE analysis (Figure 3b) based on 62 outlier loci showed that genetic clusters more clearly partitioned Red Sea and Mediterranean sampling sites compared to the analysis based on all loci (Figure 3a). Mediterranean populations, however, did not form genetic clusters based on sampling locations. In contrast, the DAPC analysis fully resolved each locality that was sampled (Figure 3d).

### 3.7 Functional analyses

Considering that SbfI (the enzyme used in this study) cuts on average every 65,000 bp (SbfI recognizes a sequence of eight nucleotides, 4^8^ = 65,536), a distance that is more than the size of a typical gene and that approximately one to five percent of the genome corresponds to protein‐coding regions, we expected for most RAD seq markers not to be found in protein‐coding regions. When comparing outlier loci with GenBank, we found that out of 62 outlier sequences, 51 had GenBank matches comprising 20 sequences that matched known protein‐coding genes (corresponding to 32% of the outlier loci), all of which being from fish species (Table S1). A coarse classification of proteins was done using a KEGG analysis. We found that 16 of the 20 recognized proteins could be classified in five different functional groups, and more than three quarters (81%) of the sequences were included in only three functional groups (genetic information processing – 31%, signaling and cellular processing – 25%, metabolism – 25%) (Figure S3).

In general, for gene analyses, even when loci are identified, specific roles for each gene, and in particular ecological functions, are usually unknown. In this study, we have identified all the outlier genes and their putative function (Table S1). As expected, however, while we have found names and even putative functions for those genes, it is difficult to extract their adaptive significance. We did, however, found two functions that seem relevant to our study system. First, we found two genes, tmc2a, and slc22a6I, that are involved with osmoregulation. Second, we have found an outlier (stanniocalcin 2a) that has been shown to be involved with fin spine size in other fish species (Roberts Kingman et al., 2021).

## 4 DISCUSSION

The lionfish invasion of the western Atlantic by *P. volitans* (and to a lesser extent by *P. miles*) has been thoroughly studied and has revealed some specific characteristics: (1) potential evidence for hybrid vigor in invaders (Whitaker & Janosik, 2020; Wilcox et al., 2018), (2) relatively few founding individuals (approximately 100, Selwyn et al., 2017), (3) lower genetic diversity compared to the source population (Betancur et al., 2011; Pérez‐Portela et al., 2018), (4) decrease in observed heterozygosity with distance to the origin of invasion (Bors et al., 2019), (5) lack of spatially explicit metapopulation genetic structure (Bors et al., 2019), and (6) evidence of loci under selection (Burford Reiskind et al., 2019). These findings provide a set of expectations for the Mediterranean lionfish invasion.

### 4.1 Presence of hybrids

Human‐mediated introductions of species often promote secondary contacts between taxa with long histories of allopatric divergence (Viard et al., 2020) and hybridization events might favor the success of invasive species, as recently suggested for the western Atlantic lionfish invasion (Whitaker & Janosik, 2020; Wilcox et al., 2018). According to Wilcox et al. (2018), western Atlantic invasive *P. volitans* lionfish are ancestral hybrids between the sister lineages *P. miles* and *P. russelii*. In the present study, despite the reported observations of *P. volitans* individuals in the Mediterranean (Gürlek et al., 2016; Turan et al., 2017), no evidence of presence or hybridization was observed. However, we did not sample populations from Turkey, were *P. volitans* has purportedly been observed. Certainly, we cannot exclude the existence of hybridized populations due to the possible role of other vectors, primarily aquarium release (Giovos et al., 2018). Further lionfish introductions could, therefore, lead to admixture, that is, intraspecific crosses between *P. miles* belonging to distinct populations (Culley & Hardiman, 2009) or to interspecific hybridization only, although hybridization between *P. miles* and *P. volitans* has not yet been documented for the western Atlantic lionfish invasion (Burford Reiskind et al., 2019). Mediterranean lionfish surveys will need to take these possibilities into consideration.

### 4.2 Genetic diversity

Founder effects are the result of genetic drift, due to low numbers of colonizing individuals and established populations, especially those recently introduced (Lin et al., 2017; Rius et al., 2015), often characterized by reduced diversity (Sendell‐Price et al., 2021). This is what was observed for the western Atlantic lionfish (Betancur et al., 2011; Selwyn et al., 2017), where either relatively few lionfish individuals were released in the region, or that the actual number of individuals in the Atlantic invasion may have been rather large (up to more than 200 individuals) but that Allee effects may have reduced fitness, resulting in lowered genetic diversity (Selwyn et al., 2017). Such an effect has only been observed in one Lessepsian migrant fish, the bluespotted cornetfish (*Fistularia commersonii*) (Bernardi et al., 2016; Golani et al., 2007; Jackson et al., 2015; Sanna et al., 2015; Tenggardjaja et al., 2014). Rather, a lack of genetic bottleneck seems to be the rule for Lessepsian migrants (Bernardi et al., 2010; Chiesa et al., 2019). This study showed that genetic diversity was similar for populations of *P. miles* in the Mediterranean and Red Sea, hence, the establishment of lionfish populations in the Mediterranean Sea was not achieved by genetically depauperate populations. Considering the recent history of this colonization (less than 10 years) (Bariche et al., 2013), our results suggest that a large number of propagules seeded the Mediterranean invasion. It is also possible that fewer propagules entered the Mediterranean, but they may have migrated together, making their effective density higher, thus avoiding genetic bottleneck.

### 4.3 Heterozygosity trends

A study based on Caribbean *P. volitans* RAD markers obtained with SbfI single digests (i.e., the same protocol used in this study) has shown that observed heterozygosity decreases with distance from the introduction point, that is Florida (Bors et al., 2019), while both allelic richness (average number of alleles per locus) and expected heterozygosity remained steady throughout the sampled range. Similarly, this study shows tantalizing data (that need further sampling to be conclusive), where allelic richness and expected heterozygosity did not show trends, while observed heterozygosity may have decreased as the sampling locality was farther away from the Suez Canal (Figure S1). The observed heterozygosity was highest in Lebanon and decreased with increased distance from the introduction point (Port Said, the exit of the Suez Canal). Again, our results were biased because our trend is mostly driven by only two influential points (those in the source). Therefore, these data should be considered as preliminary and in necessity of further sampling. If confirmed, this trend meets dispersal expectations, since genetic diversity usually declines across the expanding range of an exotic species due to genetic drift (Sendell‐Price et al., 2021).

The Lessepsian bioinvasion offers a relatively simple model system to study biological invasions because the origin of the invasion is known, the Reds Sea via the Suez Canal. The problem, however, is that the Suez Canal might provide a continuous and endless amount of novel introductions, potentially confusing genetic signals. We used samples that were obtained very early after the lionfish establishment, possibly explaining why the invasion signature, a relationship between distance and observed heterozygosity, is still visible. It is to be mentioned that repetition of this investigation during a later stage of the invasion process would be highly relevant to reveal if the genetic pattern of *P. miles* vary with regard to its residence time and propagule pressure.

In addition, the decrease occurred at a faster rate than in the Caribbean, and starting from a higher overall heterozygosity (Figure 3). This trend, however, needs to be evaluated carefully, because unlike the Caribbean study, we had few localities and smaller distances, thus reducing statistical power.

### 4.4 Population structure within the Mediterranean

The Atlantic populations of *P. volitans* lack structure, and this is mostly mirrored by Mediterranean populations of *P. miles*. Indeed, *F*_st_ values are very low (0.044) and STRUCTURE plots did not reveal obvious partitions. However, DAPC analyses do separate samples collected in Lebanon from other Mediterranean samples (based on neutral loci), suggesting that some low level of population structure does exist in the Mediterranean Sea. The fact that Lebanon is located in the easternmost sector of the basin, the first place where Lessepsian invaders establish stable populations (Golani, 2010), might explain the higher observed heterozygosity, due to multiple invasions that have reached that region but not yet spread any further, thus resulting in a Lebanese population as an independent cluster by DAPC analysis. In addition, the two other Mediterranean localities are both islands (Rhodes and Cyprus), potentially reducing immigration capability and strengthening the signal of drift.

### 4.5 Natural selection and local adaptation

When identifying genes under potential natural selection, one needs to be cautious not to exclusively focus on the few genes that seem to fit our expectations and disregard other genes with unknown functions. In our study, we have found 20 genes potentially under selection. Of those 20 genes, we were able to classify in broad categories 16 of them, and for three of them, we were able to find specific functions that seem relevant to our study. While we are discussing these three genes, it is important to appreciate that the majority of the outliers may play a greater role, but our present lack of understanding of gene functions precludes us from commenting further on those.

Recent modeling approaches identified water salinity among the most important variables explaining the distribution of Lessepsian species in the Mediterranean Sea (Belmaker et al., 2013; D'Amen & Azzurro, 2020; Parravicini et al., 2015). In previous studies, we found that genes involved with osmoregulation were under selection in invading Lessepsian fishes (Azzurro et al., 2022; Bernardi et al., 2016). Similarly, in this study, we found that two proteins are involved with osmoregulation (tmc2a, and slc22a6I). Therefore, a pattern is potentially emerging of traits that are important and shared among Lessepsian invaders, where the capability for osmoregulation might be a key factor in the success of an invasion. It has been argued that finding genes under selection in the invading range, shortly after the invasion occurred (which is the case here) is unlikely due to selection in situ, but rather evidence of preadaptation, where only those individuals that carry adaptive loci are successful invaders. This might also explain why it took so long (more than 100 years after the opening of the Canal), for lionfishes to invade. A combination of suitable ecological conditions in the Mediterranean Sea and the invasion by preadapted individuals might explain this delay.

A third gene, stanniocalcin 2a, identified as an outlier, may have an adaptive role in the context of lionfish invasion. Stanniocalcin 2a has been identified in sticklebacks as playing a role in spine development and size (Roberts Kingman et al., 2021), with distinct alleles found in short‐ and long‐spined populations. There, differential expression of the bone growth inhibitor gene Stanniocalcin2a in developing spines modifies dorsal and pelvic spine lengths in freshwater versus marine sticklebacks, which exhibit different spine length in different habitats. It is possible that lionfish might display differences in fin spine sizes in the presence and absence of predators. While at this point native and invasive lionfish spine morphological data are lacking, this is a hypothesis that may be tested in light of these findings.

## 5 CONCLUSION

Biological invasions are ecologically and economically disastrous, and their frequency are likely to increase in the near future. The study of invasion dynamics may offer key information to mitigate their impacts and to understand the complex consequences on the distribution of biological diversity; Lessepsian bioinvasions provide a unique opportunity to do so. It is particularly interesting to compare biological invasions, yet this is rarely done because each invasion has its own characteristics (Azzurro et al., 2022; Bernardi et al., 2016). The lionfish invasions in the Atlantic and the Mediterranean Sea show some parallels but also some marked differences. For example, both invasions may show a decrease in observed heterozygosity with distance from the invasion source. Both also show evidence of natural selection that could potentially identify key genes related to invasion success. Yet, there are some important differences. The main one being that Atlantic invaders are genetically depauperate, while Mediterranean invaders do not show a lowered genetic diversity compared to the source population. This is likely due to the different modes of invasion in the two systems.

Since the opening of the Suez Canal in 1869, hundreds of Red Sea Lessepsian invaders entered the Mediterranean Sea. There is no arguing that Lessepsian migrations are an artificial phenomenon, yet, once the Canal was opened, Red Sea species moved along this route by active (swimming individuals) or passive (larval drift) spread, without other direct human assistance. The construction of the Aswan Dam in 1960 decreased the freshwater outflow of the Nile River, removing one of the most important obstacles to Lessepsian migration, the widening of the Suez Canal has decreased the effectiveness of the bitter lakes as an ecological barrier (Galil et al., 2015), and climate change has increased the overall suitability of the Mediterranean Sea for Lessepsian invaders (D'Amen & Azzurro, 2020). The 193 km long Suez Canal is possibly acting as both an invaded ecosystem and a pathway through which dispersing pioneers advance toward the Mediterranean Sea using it as a stepping stone (Azzurro et al., 2022). Other habitats, such as sandy areas in the northern Red Sea, have also been proposed as launching pads to successful invasion bouts (Golani, 1993). This is fundamentally different from human‐mediated transport of individuals, such as via ship ballast water, or aquarium releases, such as for the western Atlantic lionfish invasion. In the former, species life histories and genetic characteristics play an important role in providing individuals or populations the capability of breaching the barrier, while in the latter the chance of transport is the main factor in the invasion success. Such differences result in very different genetic signatures. The vast literature on the Atlantic invasion lays the groundwork to compare it with the Mediterranean. In the Atlantic, a relatively small number of individuals were introduced (however, many individuals were released from private aquariums), while in the Mediterranean Sea, only individuals that had adequate fitness, or that were selected for specific traits (e.g., genes related to osmoregulation) during their permanence in the Suez Canal, could successfully colonize the Mediterranean Sea. Once ready to invade, individuals could successfully adapt and reproduce, resulting in high propagule pressure and high observed heterozygosity as a consequence. Overall, the Mediterranean lionfish invasion gives a great opportunity to test general hypotheses in invasion biology and range expansions in general. Some classic examples of natural and mediated introductions have also provided interesting models to identify specific predictions of range expansions. Monarch butterflies (*Danaus plexippus*) and silvereye birds (*Zosterops lateralis*) present ideal range expansions worldwide, and in the Pacific Islands, respectively, to test such predictions (Pierce et al., 2014; Sendell‐Price et al., 2021). Future genomic approaches using a large number of Lessepsian migrants will allow to test specific range expansion models and help determine the ecological and evolutionary trends of successful invaders, the role of preadaptation and which selected genes are shared among different successful invading species.

## AUTHOR CONTRIBUTIONS

**Giacomo Bernardi:** Conceptualization (equal); data curation (equal); formal analysis (equal); investigation (equal); writing – original draft (equal); writing – review and editing (equal). **Ernesto Azzurro:** Conceptualization (equal); formal analysis (equal); investigation (equal); methodology (equal); validation (equal); writing – review and editing (equal). **Michel Bariche:** Conceptualization (equal); data curation (equal); formal analysis (equal); investigation (equal); writing – review and editing (equal). **Carlos Jimenez:** Conceptualization (equal); data curation (equal); formal analysis (equal); investigation (equal); writing – review and editing (equal). **Stefanos Kalogirou:** Conceptualization (equal); data curation (equal); formal analysis (equal); investigation (equal); writing – review and editing (equal). **Periklis Kleitou:** Conceptualization (equal); data curation (equal); formal analysis (equal); investigation (equal); writing – review and editing (equal).

## FUNDING INFORMATION

Funding for this work was provided by the Committee on Research, COR‐UCSC, to GB, by the LIFE financial instrument of the European Union—RELIONMED project (Grant Agreement LIFE16 NAT/CY/000832) to PK, ENALIA's Research Program, and by the University Research Board of the AUB (DDF 103367/23927) to MB.

## Supporting information


Figure S1

Figure S2

Figure S3

Table S1

Data S1

## Data Availability

All Fastq sequence files are available from GenBank at the National Center for Biotechnology Information short‐read archive database (accession number: SAMN40146221‐SAMN40146337). Associated metadata are also available at GeOMe (https://n2t.net/ark:/21547/FFt2).
