# Supplementary figures and images for: Invasion genomics of lionfish in the Mediterranean Sea
Source: Ecol Evol. 2024 Mar 5;14(3):e11087. doi: 10.1002/ece3.11087 (PMC10915480; doi:10.1002/ece3.11087)

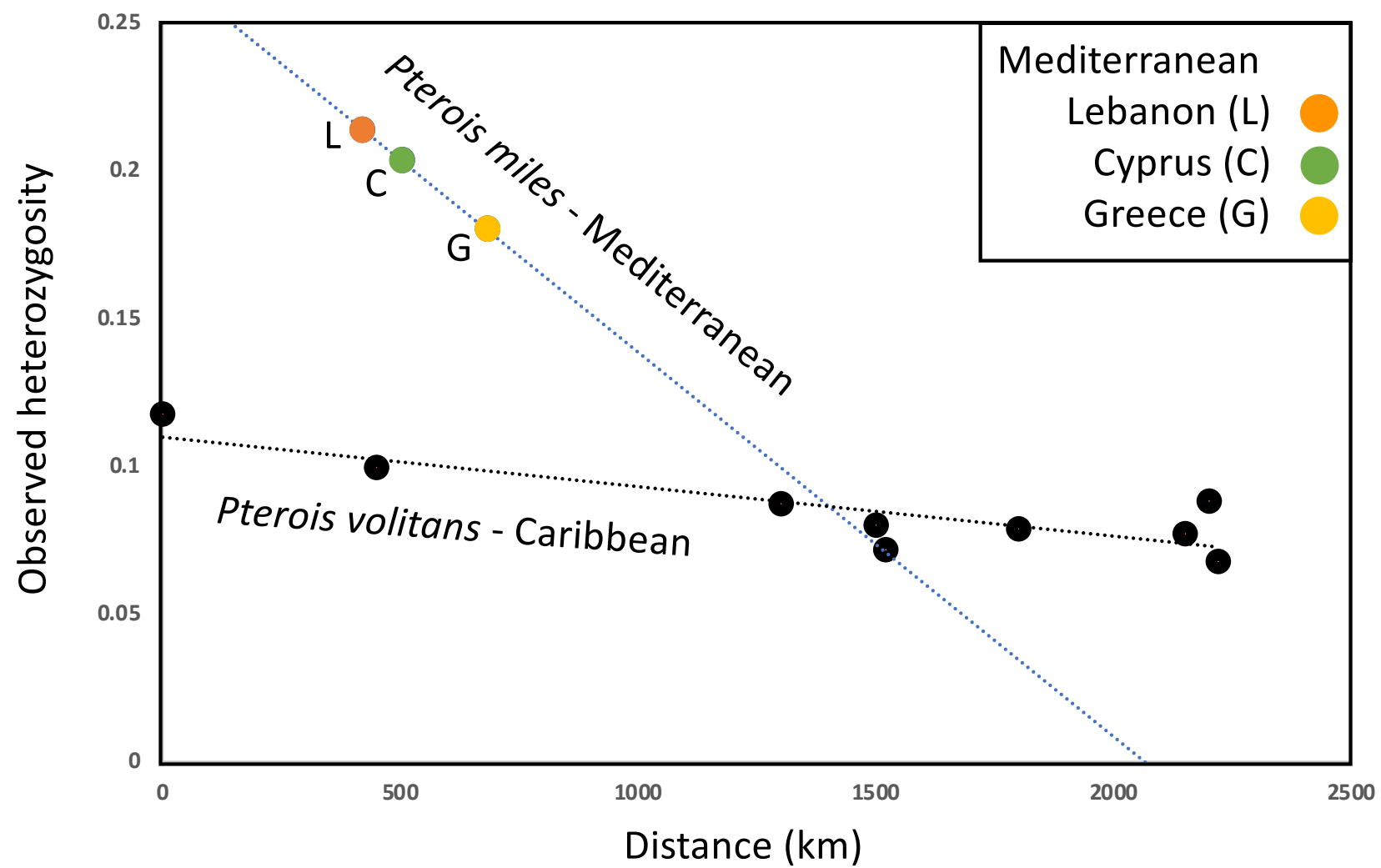

Figure\_S1

Supplement: Supplementary file 1 — Figure S1 [file ECE3-14-e11087-s001.pdf]

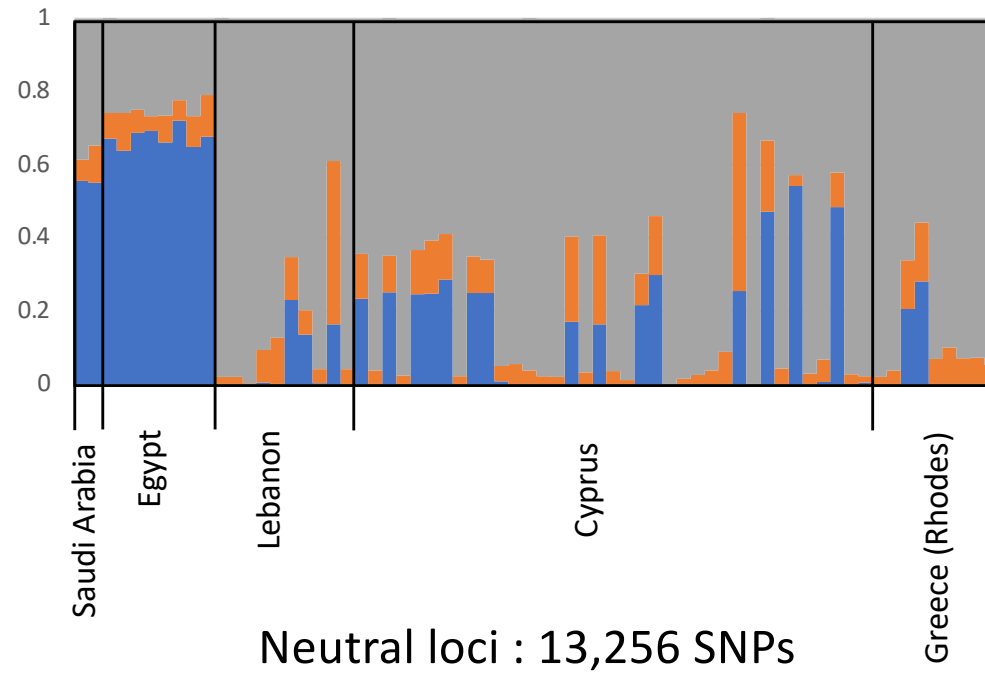

Figure S2

Supplement: Supplementary file 2 — Figure S2 [file ECE3-14-e11087-s004.pdf]

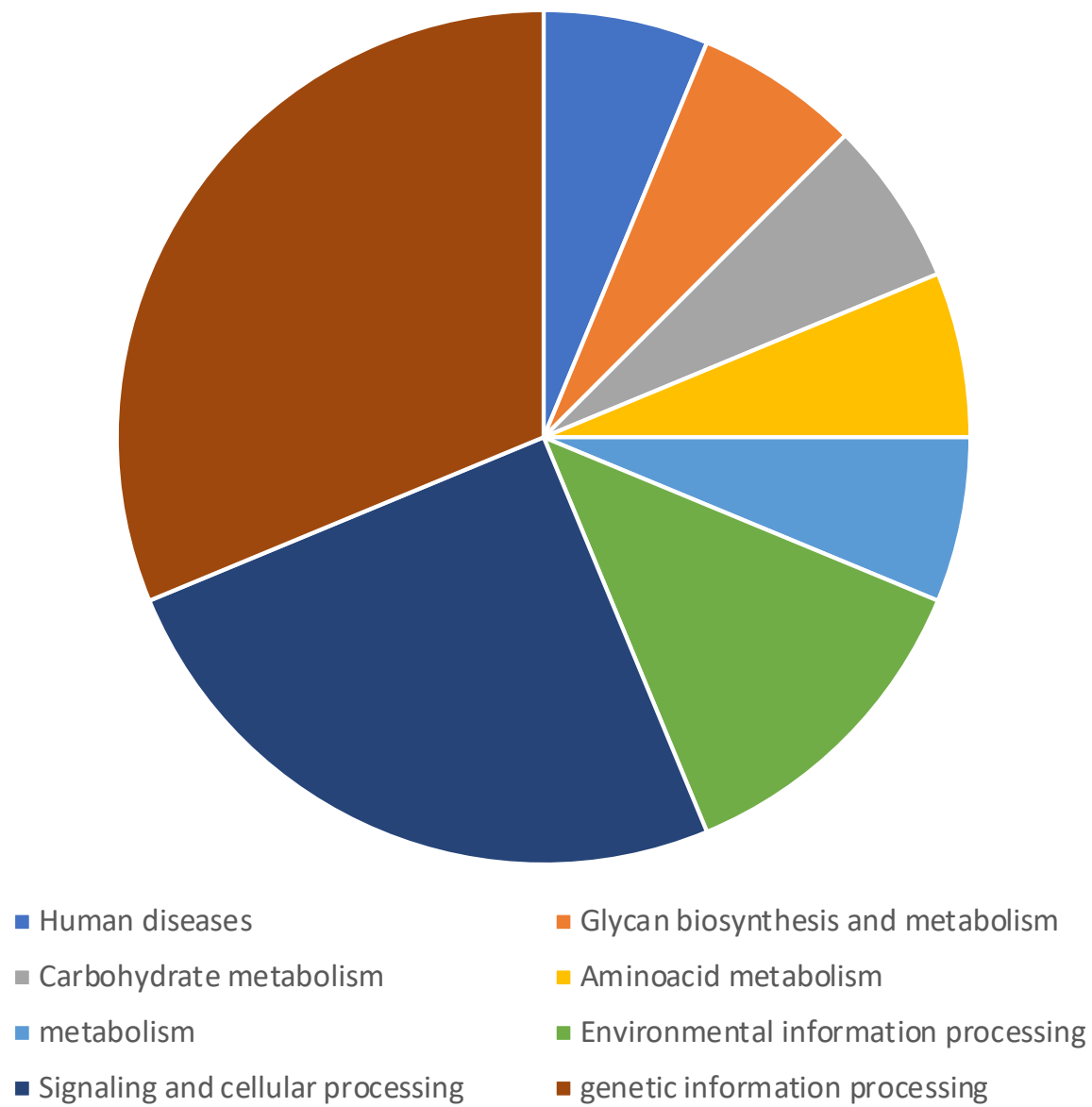

Figure S3

Supplement: Supplementary file 3 — Figure S3 [file ECE3-14-e11087-s002.pdf]
