# Supplementary material for: Invasion genomics of lionfish in the Mediterranean Sea
Source: Ecol Evol. 2024 Mar 5;14(3):e11087. doi: 10.1002/ece3.11087 (PMC10915480; doi:10.1002/ece3.11087)
Supplement: Supplementary file 4 — Table S1 [file ECE3-14-e11087-s005.docx]

Supplementary table S1

GenBank results of genes blast matching outlier loci of *Pterois miles.* The first column corresponds to GenBank accession numbers, the second column corresponds to gene identifiers

XM_037748879 Sebastes umbrosus alpha-1,3-mannosyl-glycoprotein 4-beta-N-acetylglucosaminyltransferase C-like sugar metabolism

XM_032511064 Etheostoma spectabile transcobalamin-1-like  digestion

XM_037753412 Sebastes umbrosus transmembrane channel-like 2a (tmc2a) **ion channel activity**

XM_037755611 Sebastes umbrosus phosphoglycolate phosphatase cofactor of the glycolytic enzyme

XM_037780859 Sebastes umbrosus stanniocalcin 2a (stc2a) **Fin spine lengths**

XM_035998121 Sander lucioperca RNA-binding Raly-like protein Immune response

XM_034555701 Cyclopterus lumpus zonadhesin-like (LOC117746517) gamete recognition

XM_037791001 Sebastes umbrosus protein Wiz (si:ch211-194b1.1), transcript variant X3 zinc finger

XM_037748879 Sebastes umbrosus solute carrier family 22 member 6, like (slc22a6l) **renal organic anion transport**

XM_031868089 Anarrhichthys ocellatus kelch like family member 36 (klhl36), transcript variant X4 cell function

XM_028591006 Perca flavescens obscurin-like protein 1 (LOC114563952), transcript variant X14 cell function

XM_028587302 Perca flavescens DENN domain containing 1B (dennd1b) guanine nucleotide exchange factor

XM_034547937 Cyclopterus lumpus WD repeat domain 81 (wdr81) role in mitosis

XM_037758019 Sebastes umbrosus exocyst complex component 6B (exoc6b), transcript variant X1 role in exocytosis

 XM_042483870 Plectropomus leopardus methylcrotonyl-CoA carboxylase protein metabolism

XM_030066616  Myripristis murdjan myosin XVIIIA (myo18a), transcript variant X4 intracellular transport

XM_033644395 Epinephelus lanceolatus probable G-protein coupled receptor 160 (si:dkeyp-100a1.6) role in hormone response

XM_037789453 Sebastes umbrosus glycine receptor, alpha 2 (glra2) chloride channel

XM_037786904 Sebastes umbrosus MIB E3 ubiquitin protein ligase 1 (mib1) ligand of Notch proteins

XM_033620410 Epinephelus lanceolatus ral guanine nucleotide dissociation stimulator-like (LOC117253070) role in size differntiation
