# Supplementary material for: Invasion genomics of lionfish in the Mediterranean Sea
Source: Ecol Evol. 2024 Mar 5;14(3):e11087. doi: 10.1002/ece3.11087 (PMC10915480; doi:10.1002/ece3.11087)
Supplement: Supplementary file 5 — Data S1 [file ECE3-14-e11087-s003.pdf]

Mediterranean_lionfish

Giacomo Bernardi

1/4/2023

This tutorial explains how we cleaned our RAD sequences following Michael Miller's (UC Davis) protocol

Quality filter of the raw sequences

```
perl QualityFilter_ready96.pl File.txt > File_qf.txt
```

QualityFilter_ready96.pl is as follows

```
#!/usr/bin/perl;
##########################################################################
$percent_filter = 80;
$length = 96;
$phred = 33;  #The value should be either 33 or 64 depending on the base-calling pipelin
e.
##########################################################################


$file = $ARGV[0];

open(FILE, "File.txt")
    or die;

while (<FILE>) {

    $R1_ID1 = $_;
    $R1_seq = <FILE>;
    $R1_ID2 = <FILE>;
    $R1_qual = <FILE>;
    chomp($R1_seq);
    chomp($R1_qual);

    $R1_seq = substr($R1_seq,0,$length);
    $R1_qual = substr($R1_qual,0,$length);

    if ($R1_seq =~ m/N/) {

    } else {

        @ASCII = unpack("C*", $R1_qual);
        $prob = 100;
        $x = 1;
        $R1_length = length($R1_qual);
        foreach $value (@ASCII) {
            $value = $value - $phred;
            $prob = $prob * (1-(10**(-$value/10)));
            $x++;
        }

        if ($prob >= $percent_filter) {
            $good_reads++;
            print $R1_ID1;
            print "$R1_seq\n";
            print $R1_ID2;
            print "$R1_qual\n";
        }

    }
```

```
}
close FILE;
```

sequences are clean, now we need to demultiplex in 96 different files.

place three files in the same folder: sbfi_barcodes.txt, run_96.sh, BarcodeSplit_96.pl

```
sh run_96.sh
```

(this is a weird very short command, but it works)

if you look at the sbfi_barcodes.txt file, you will see that each lane is made of GG then the barcode and then 6 letters. This is the prefix of every sequence, we will identify this pattern and remove it. These are 16 letters, which once removed from 96bp will result in 80bp again check with text wrangler run_96.sh and BarcodeSplit_96.pl to be sure the files have the correct names.

below are the three files

run_96.sh

```
#!/bin/bash


x=1
while [ $x -le 96 ]
do

    string="sed -n ${x}p sbfi_barcodes.txt"
    str=$($string)

    var=$(echo $str | awk -F"\t" '{print $1,$2,$3}')
    set -- $var
    well=$1
    index=$2

    perl BarcodeSplit_96.pl lane1.fastq ${index} > lane1_${well}.fastq


    x=$(( $x + 1 ))

done
```

BarcodeSplit_96.pl

```
#!/usr/bin/perl;
if ($#ARGV == 1) {
    $file = $ARGV[0];
    $barcode = $ARGV[1];
} else {
    die;
}

$barcode_length = length($barcode);

open(FILE, "GB051702_qf.txt")
    or die;

while (<FILE>) {

    $ID_line_1 = $_;
    $seq_line = <FILE>;
    $ID_line_2 = <FILE>;
    $Quality_line = <FILE>;

    chop $seq_line;
    $bc = substr($seq_line,0,$barcode_length);

    if ($bc eq $barcode)  {
        $correct_count++;

        $read_length = length($seq_line) - ($barcode_length);
        $read = substr($seq_line,($barcode_length),$read_length);

        $Quality_line_trimmed = substr($Quality_line,($barcode_length),$read_length);

        print $ID_line_1;
        print "$read\n";
        print $ID_line_2;
        print "$Quality_line_trimmed\n";

    }

}
close FILE;
```

sbfi_barcodes.txt

```
A01 AAACGG
A02 AACGTT
A03 AACTGA
A04 AAGACG
A05 AAGCTA
A06 AATATC
A07 AATGAG
A08 ACAAGA
A09 ACAGCG
A10 ACATAC
A11 ACCATG
A12 ACCCCC
B01 ACTCTT
B02 ACTGGC
B03 AGCCAT
B04 AGCGCA
B05 AGGGTC
B06 AGGTGT
B07 AGTAGG
B08 AGTTAA
B09 ATAGTA
B10 ATCAAA
B11 ATGCAC
B12 ATGTTG
C01 ATTCCG
C02 CAAAAA
C03 CAATCG
C04 CACCTC
C05 CAGGCA
C06 CATACT
C07 CCATTT
C08 CCCGGT
C09 CCCTAA
C10 CCGAGG
C11 CCGCAT
C12 CCTAAC
D01 CGAGGC
D02 CGCAGA
D03 CGCGTG
D04 CGGTCC
D05 CGTCTA
D06 CGTGAT
D07 CTACAG
D08 CTCGCC
D09 CTGCGA
D10 CTGGTT
D11 CTTATG
D12 CTTTGC
E01 GAAATG
E02 GAACCA
E03 GACGAC
E04 GACTCT
```

```
E05 GAGAGA
E06 GATCGT
E07 GCAGAT
E08 GCATGG
E09 GCCGTA
E10 GCGACC
E11 GCGCTG
E12 GCTCAA
F01 GGACTT
F02 GGCAAG
F03 GGGCGC
F04 GGGGCG
F05 GGTACA
F06 GGTTTG
F07 GTAAGT
F08 GTATCC
F09 GTCATC
F10 GTGCCT
F11 GTGTAA
F12 GTTGGA
G01 TAAGCT
G02 TAATTC
G03 TACACA
G04 TACGGG
G05 TAGTAT
G06 TATCAC
G07 TCAAAG
G08 TCCTGC
G09 TCGATT
G10 TCGCCA
G11 TCGGAC
G12 TCTCGG
H01 TCTTCT
H02 TGAACC
H03 TGACAA
H04 TGCCCG
H05 TGCTTA
H06 TGGGGA
H07 TTATGA
H08 TTCCGT
H09 TTCTAG
H10 TTGAGC
H11 TTTAAT
H12 TTTGTC
```
